# Vision-Based Autonomous Following of a Moving Platform and Landing for an Unmanned Aerial Vehicle

**DOI:** 10.3390/s23020829

**Published:** 2023-01-11

**Authors:** Jesús Morales, Isabel Castelo, Rodrigo Serra, Pedro U. Lima, Meysam Basiri

**Affiliations:** 1 Institute for Mechatronics Engineering & Cyber-Physical Systems (IMECH), Universidad de Málaga, 29071 Málaga, Spain; 2 Instituto Superior Técnico (IST), Universidade de Lisboa, 1049-001 Lisboa, Portugal

**Keywords:** unmanned aerial vehicle, unmanned ground vehicle, autonomous landing, target following, pose estimation, artificial fiducial markers, cascade loop

## Abstract

Interest in Unmanned Aerial Vehicles (UAVs) has increased due to their versatility and variety of applications, however their battery life limits their applications. Heterogeneous multi-robot systems can offer a solution to this limitation, by allowing an Unmanned Ground Vehicle (UGV) to serve as a recharging station for the aerial one. Moreover, cooperation between aerial and terrestrial robots allows them to overcome other individual limitations, such as communication link coverage or accessibility, and to solve highly complex tasks, e.g., environment exploration, infrastructure inspection or search and rescue. This work proposes a vision-based approach that enables an aerial robot to autonomously detect, follow, and land on a mobile ground platform. For this purpose, ArUcO fiducial markers are used to estimate the relative pose between the UAV and UGV by processing RGB images provided by a monocular camera on board the UAV. The pose estimation is fed to a trajectory planner and four decoupled controllers to generate speed set-points relative to the UAV. Using a cascade loop strategy, these set-points are then sent to the UAV autopilot for inner loop control. The proposed solution has been tested both in simulation, with a digital twin of a solar farm using ROS, Gazebo and Ardupilot Software-in-the-Loop (SiL); and in the real world at IST Lisbon’s outdoor facilities, with a UAV built on the basis of a DJ550 Hexacopter and a modified Jackal ground robot from DJI and Clearpath Robotics, respectively. Pose estimation, trajectory planning and speed set-point are computed on board the UAV, using a Single Board Computer (SBC) running Ubuntu and ROS, without the need for external infrastructure.

## 1. Introduction

In recent years, unmanned aerial vehicles (UAVs) have driven some of the most important sectors of the economy. Their benefits are hard to ignore, and their versatility makes them suitable for almost every industry. UAVs, easily equipped with cameras and range sensors, can cover large areas in short periods of time while inspecting, recording and building maps. Sectors such as construction [1,2] use UAVs for asset monitoring, while surveillance applications [3,4] focus on autonomous monitoring of homes and businesses. In the field of conservation and exploration, there are projects using drones to monitor the natural environment and wildlife [5] or to discover the extent of ancient buried civilizations [6].

The integration of robots into the renewable energies sector has also been growing [7]. Given the alarming concerns around global warming, researchers are now looking at ways to reduce costs and accelerate the performance of wind and solar plants. In this direction, the DURABLE project [8] considers the collaboration of a heterogeneous multi-robot system to automate solar panel inspection and repair tasks. In this joint project, a subset of UAVs provides fast inspection of the solar plant, while Unmanned Ground Vehicles (UGVs) work as inspector of individual solar panels as well as charging station for the UAVs. This functionality requires the UAV to autonomous follow and land on the UGV.

This paper proposes a vision-based system in which a UAV can autonomously follow a moving UGV, and enable the UAV to land on a UGV serving as a landing platform. In this way, the main contributions are the following:

- A custom-designed landmark pattern composed of ArUCo markers [9,10] and a method to estimate the UAV relative position and heading w.r.t. the UGV is presented.
- A hierarchical controller for the following and the landing that runs onboard the UAV and that exclusively relies on markers is developed. Concretely, an Ardupilot flight controller is employed as autopilot at the low-level, and a single-board computer (SBC) implements a trajectory tracking high-level controller for relative 3D position and heading, with a trapezoidal profile speed generator as feedforward and four decoupled PI controllers in the feedback loop.
- Tests using a realistic heterogeneous multi-robot simulator as well as in real-world outdoor scenario are presented.

The rest of the paper is organised as follows. Section 2 highlights the main contributions of the work with respect to the most related studies. Then, Section 3 describes the aerial and ground vehicles used in this work as well as the simulation tools and auxiliary equipment. In Section 4 the autonomous following and landing system is presented. Simulated and real experiments are discussed in Section 5. Finally, conclusions, acknowledgements, and references complete the paper.

## 2. Related Work

Previous work in this area has explored different markers and control strategies to safely perform the task of following and landing a UAV. Works such as Baca et al. [11], Falanga et al. [12] have used a custom landmark represented by a crossed circle surrounded by a rectangle, and rely on range finder sensors, whereas Polvara et al. [13] used only the crossed circle as a reference point.

In the marker processing state, Baca et al. [11], Falanga et al. [12] apply adaptive thresholds in which the shapes are detected in a predefined order and matched against the previously known standards. Past this stage, Baca et al. [11] follows a Model Predictive Control (MPC) strategy to track the moving target whilst a commercial flight controller provides the measurements regarding the UAV position, velocity and orientation. The latter are corrected by a differential RTK (Real Time Kinematic) GPS using LKF (Linearized Kalman filter) fusion as well as the vertical position estimate, assisted by a TeraRanger range finder and the landmark detection algorithm.

On the other hand, Falanga et al. [12] follows a non-linear control strategy that drives the quadrotor forward towards the desired trajectory using a high and low-level controller. The high-level controller takes the difference between the reference and estimated position, velocity, acceleration and jerk as inputs and returns the derived collective thrust and body rotations. The low-level controller takes the outputs of the high-level controller and computes the necessary torques to apply to the rigid body. The work developed in Polvara et al. [13] has been tested only in simulation and it takes a slightly different approach as it implements a hierarchy of Deep Q-Networks (DQN) for each step of the landing phase: landmark detection, descend manoeuvre, and touchdown. It also uses a PID (Proportional–Integral–Derivative) controller to assist the final touchdown manoeuvre.

In Lange et al. [14], the authors use a sequence of rings surrounded by a hexagonal shape as the reference marker. The visual tracking system identifies the landing pad through the unique radius of each circle, making it distinguishable at high and low altitudes. The marker detection relies on image segmentation with a fixed threshold and in image invariant moments. Regarding the control actuation, the algorithm starts by correcting the measurement of the distance to the landing pad with the current pitch and roll angles. Following this step, a PID controller takes these corrections and computes the necessary motion commands to keep the UAV steady above the centre of the landing pad. In their setup, a ground station is required to process the images from the onboard camera, to run the PID control loop and to generate the necessary motion commands.

In this line of thought, the work developed in Lee et al. [7], Hui et al. [15], Cabrera-Ponce and Martinez-Carranza [16] also use custom markers for detection. Hui et al. [15] employs a white circle with a 20 cm radius, Cabrera-Ponce and Martinez-Carranza [16] relies on a flag and H-shaped tag, whereas Lee et al. [7] focuses on a red rectangle placed on top of the moving target.

Although tested and proven with quality results, custom markers make the whole detection, tracking and landing algorithm computationally more expensive. Compared to fiducial tags, available off the shelf as open-source algorithms, the entire procedure becomes harder to implement.

To the knowledge of this work, there are different kinds of fiducial markers, among which ARTag, AprilTag, ArUcO and STag are the most common [17]. Works developed by Delbene et al. [18] and Gautam et al. [19] use AprilTags to assist the landing, whereas Chang et al. [20] relies on ArUco tags.

Delbene et al. [18] proposes a methodology that estimates the target’s relative pose and velocity, employing not only AprilTags on the landing platform but also ultrasonic sensors on the UAV. According to these authors, ultrasonic sensors added robustness during the final landing phase, given the unreliability of the measurements achieved with AprilTags. Although tested under simulation with the recreation of realistic behaviours of the landing platform, the work does not present tests done in a real-world marine environment. Moreover, the ultrasonic sensor only provides the altitude, and it is often unreliable due to their small field of view. This constraint leads to a poor cost-performance trade-off, given that the sensory system has another input to process. As presented in our work, the markers should be enough to estimate the UAV relative position as well as the heading.

Gautam et al. [19] addresses the same problem by proposing a vision-based guidance approach with a log-polynomial closing velocity controller integrated with pure pursuit guidance. In their work, the landing pad detection algorithm uses a combination of colour segmentation and AprilTags to ensure flexibility and detectability from low and high altitudes. For better altitude estimates, the authors have also used a LiDAR. In this work, the vision pipeline chooses a random AprilTag as the landing target centre, which it keeps tracking during the landing phase. If the camera system loses this marker, the algorithm initializes the tracking algorithm with a new randomly selected AprilTag. This idea seems rather unusual as it focuses on one randomly selected tag at the time. Furthermore, the approach focuses on AprilTags to assist in the landing, but it does not use them for pose estimation.

The work of Chang et al. [20] proposes an autonomous landing system based on the implementation of a ground-effect trajectory. Regarding the UAV position estimation, the work exploited a sensor fusion-based algorithm based on a Kalman filter. The estimation method used Inertial Measurement Unit (IMU) data, stereo depth information, ArUco markers and YOLO object detector. Although it focuses on minimizing the demand for the UAV payload whilst maximizing the usage of the computational power, having the computation unit exclusively located on the ground vehicle seems feasible for landing purposes but not achievable in application cases.

Rodriguez-Ramos et al. [21] developed a deep reinforcement learning strategy for autonomous UAV landing on a moving platform. The work focuses on indoor scenarios, employing an Optitrack motion capture system (Mo-cap) to accurately localise both vehicles, as well as a workstation to implement the UAV controller and command it through a wireless link.

## 3. System Description

**Figure 1:**
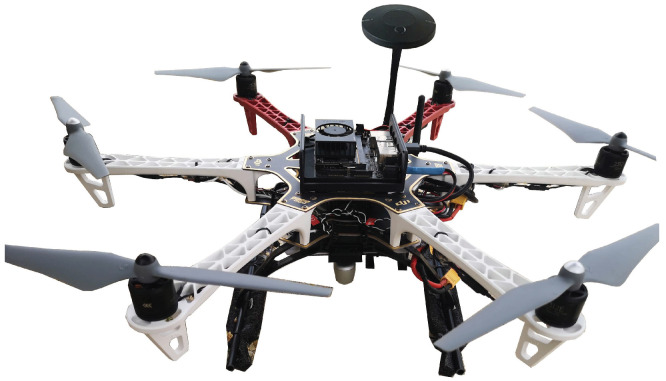
Unmanned aerial vehicle based on a DJI F550 hexacopter.

The presented work has been tested in a system consisting of a drone and a ground vehicle. The aerial platform is based on a DJI F550 hexacopter (see Figure 1). It is equipped with a Hex Cube Black flight controller with a vibration damped IMU and a Here+ GNSS receiver. It runs Ardupilot and provides takeoff functionality and a *guided* mode to externally control the drone horizontal location, altitude and heading [22]. A Jetson Xavier NX onboard computer with 6-core ARM CPU, 284-core NVIDIA GPU, and 8 GB RAM running Ubuntu and ROS is used for high level tasks, including pose estimation and speed set-point generation to command the drone. Communication between the flight controller and the companion computer is achieved using a serial interface and MAVROS, a MAVLink-to-ROS gateway with proxy for Ground Control Station [23]. Images are provided by the $69{}^{\circ }\times 42{}^{\circ }$ field-of-view RGB monocular camera of an Intel Realsense 435 device mounted on board the drone. The UAV is powered by two 14.8 V lithium polymer (LiPo) batteries connected in parallel with a total capacity of 8000 mAh, allowing a flight time between 12 min and 15 min.

**Figure 2:**
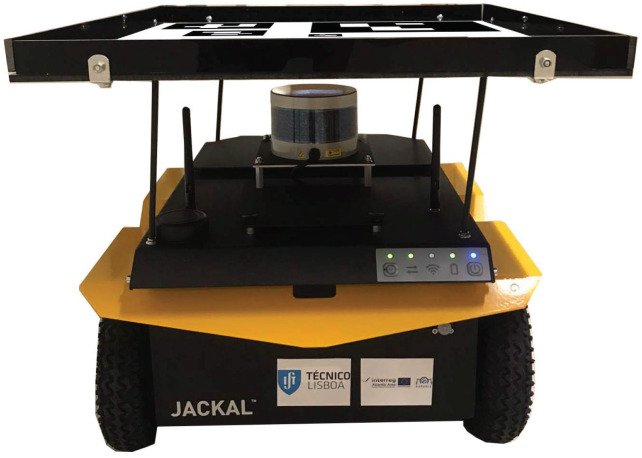
Jackal mobile robot with landing platform and marker pattern on top.

The ground vehicle is a modified Jackal mobile robot from Clearpath Robotics. A 50 cm width and 56 cm long landing platform with a marker pattern has been added on top as shown in Figure 2. Jackal can perform way-point navigation as well as being teleoperated via a wireless gamepad.

A laptop computer with a gamepad was also employed during the tests. It connected wirelessly to the UAV onboard computer and allowed performing tasks such as:

- UAV initialization and mode selection (teleoperated or autonomous),
- UAV teleoperation via the gamepad, or
- sending relative pose set-points or landing commands.

**Figure 3:**
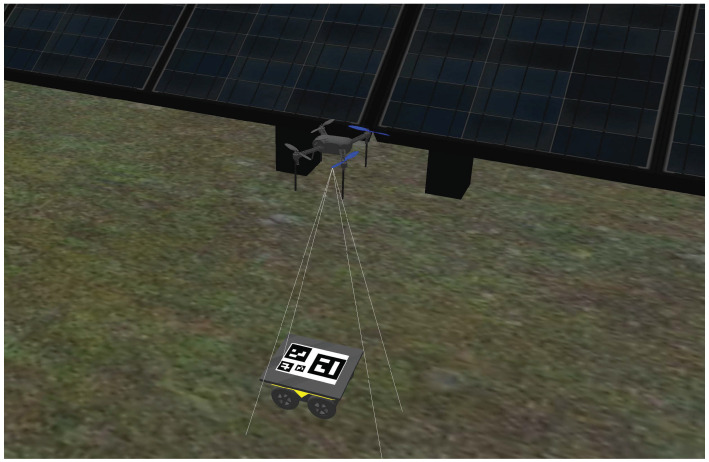
Simulation of a modified Jackal and a quadcopter with an onboard camera.

The Gazebo environment developed for simulation purposes is a digital twin of the actual solar farm used in the real-world tests for the DURABLE project. The simulation aggregates multiple ROS packages from which multi-jackal, ardupilot and ardupilot-gazebo are the most relevant. These packages support multiple modified Jackal robots and a quadcopter with an ardupilot flight controller and an onboard RGB camera that enable Software-in-the-Loop (SiL) simulations [24] (see Figure 3).

## 4. Autonomous Following and Landing

**Figure 4:**
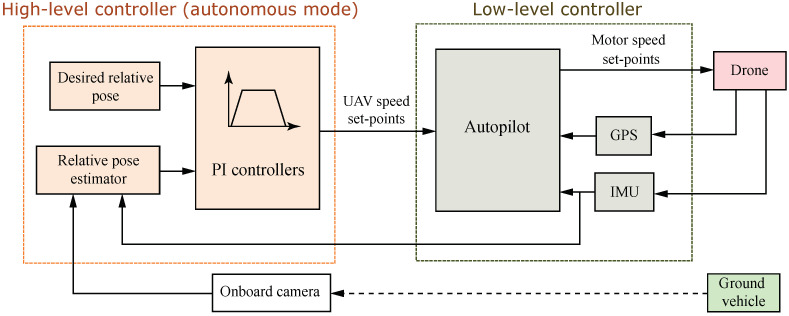
UAV hierarchical system diagram.

This work presents a vision-based hierarchical system that allows an aerial robot to follow and land autonomously on a ground mobile platform (see Figure 4). To this end, fiducial markers are used to estimate the relative pose between the UAV and UGV. The high-level controller has two operation modes: autonomous and teleoperated. In autonomous mode pose estimations are fed to a trajectory planner and four decoupled controllers to generate speed set-points relative to the stabilized UAV reference frame in order to follow the UGV or land on it. Using a cascade loop strategy, these set-points are then sent to the UAV autopilot for inner-loop control. In teleoperation mode, speed set-points are received directly from a ground station and sent to the low-level controller.

### 4.1. Relative Pose Estimation

Relative pose estimation between the UAV and the UGV is done by localizing a set of ArUcO markers placed on top of Jackal landing platform. ArUcO makers were chosen from others fiducial markers such as ARTag, STag or AprilTag because its low computational cost and good precision considering the comparison presented in [17].

**Figure 5:**
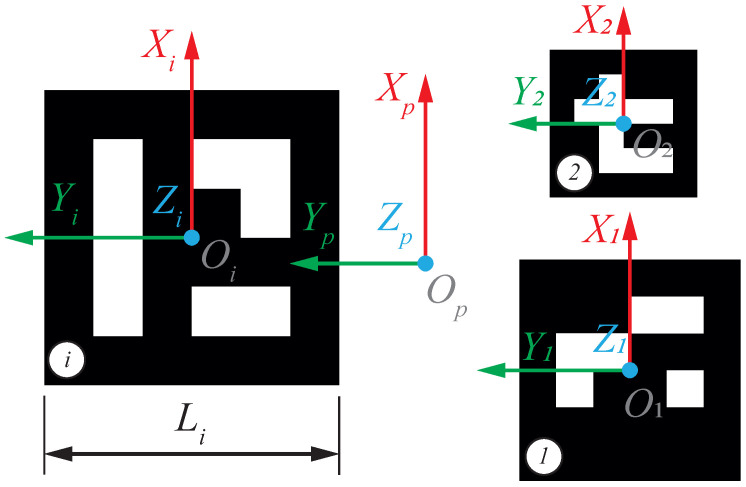
Reference frame definition for a set of ArUcO markers.

Each marker has associated an identification code *i*, a side length $L_{i}$, and a reference frame $XYZ_{i}$ as shown in Figure 5. An additional reference frame $XYZ_{p}$ is defined for the marker pattern. Markers are added to the pattern plane without rotating, so the transformation ${}^{p}T_{i}$ from each marker frame *i* to the pattern frame *p* is determined by the translation

(1) $${}^{p}t_{i}={\left(\begin{array}{ccc} {}^{p}x_{i} & {}^{p}y_{i} & 0 \end{array}\right)}^{T},$$

where ${}^{p}x_{i}$ and ${}^{p}y_{i}$ are the coordinates of pattern frame origin $O_{p}$ w.r.t. the maker frame *i*.

The position and the orientation (quaternion) of each marker in the camera frame $XYZ_{c}$ is computed using the aruco_detect node from the fiducial ROS package [25]. Two additional right-handed reference frames are considered: body and stabilized. The first one, $XYZ_{b}$, is attached to the UAV, with the *X* and *Y* axes pointing in the drone forward direction and to the left, respectively. The second one, $XYZ_{s}$, has the same origin and heading that the body frame but the plane defined by its *X* and *Y* is parallel to the ground. The relation between the camera and the body frames is a fixed transformation, ${}^{b}T_{c}$. But the one between the body and the stabilized frame, ${}^{s}T_{b}$, varies according to the pitch and roll angles of the drone. This has been computed using the measurements provided by the IMU flight controller via MAVROS and then hector_imu_attitude_to_tf ROS node [26].

Each detected marker *i* provides an estimation of the pattern frame pose with respect to the stabilized frame, ${}^{s}{\hat{T}}_{pi}$. Its Cartesian coordinates can be obtained by averaging individual estimations, and weighting the altitude component with the detected marker area, $A_{i}$, for robustness:

(2) $${}^{s}{\hat{x}}_{p}=\sum_{i}{}^{s}{\hat{x}}_{pi},{\;}^{s}{\hat{y}}_{p}=\sum_{i}{}^{s}{\hat{y}}_{pi},\;{}^{s}{\hat{z}}_{p}=\frac{1}{A}\sum_{i}{}^{s}{\hat{z}}_{pi}A_{i},$$

where $A=\sum_{i}A_{i}$. The estimated orientation of the pattern frame w.r.t. the stabilized frame in quaternion form, ${}^{s}{\hat{q}}_{p}$, can be obtained from quaternion averaging using the eigendecomposition method presented in [27]. However, if quaternions are close to each other, as is in this case, element-wise averaging followed by normalization produce much faster estimations [28]. Additionally, the double-cover issue, i.e., q and −q representing the same rotation, need to be taking into account. This can be done by choosing one of the estimations as reference, ${\hat{q}}_{r}$, and negating each quaternion ${\hat{q}}_{i}$ whose scalar product with ${\hat{q}}_{r}$ is negative. As long as bigger markers provide more precise estimation, the maker area $A_{i}$ is considered in obtaining the averaged estimation:

(3) $${}^{s}{\hat{q}}_{p}=\frac{{}^{s}{\bar{q}}_{p}}{|{}^{s}{\bar{q}}_{p}|},\;{}^{s}{\bar{q}}_{p}=\frac{1}{A}\sum_{i}{}^{s}{\hat{q}}_{pi}A_{i},$$

where ${}^{s}{\hat{q}}_{pi}$ is the orientation, in quaternion form, of the platform frame w.r.t. the stabilized frame estimated using marker *i*, and taking into account the double-cover issue. The estimated yaw angle $\hat{\psi }$ can be obtained using the function getRPY from the tf2 library [29]. Then, the estimated relative pose of the pattern frame w.r.t. stabilized body frame is defined as

(4) $${}^{s}{\hat{p}}_{p}={\left({}^{s}{\hat{x}}_{p},\;{}^{s}{\hat{y}}_{p},\;{}^{s}{\hat{z}}_{p},\;\hat{\psi }\right)}^{T}.$$

### 4.2. Speed Set-Point Generation

**Figure 6:**
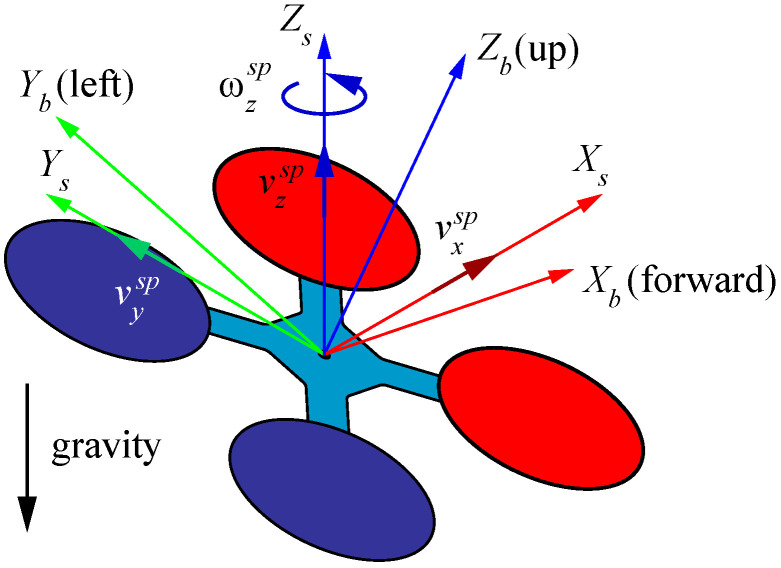
UAV Body and stabilized reference frames.

The UAV flight controller is configured to operate in *guided* mode for autonomous following and landing. This allows the onboard computer to control the horizontal position, altitude and heading of the UAV by sending speed set-point to the autopilot through MAVROS. Specifically, three linear and one angular speed set-points relative to the stabilized UAV reference frame are commanded, represented by $v^{sp}={\left(v_{x}^{sp},\;v_{y}^{sp},\;v_{z}^{sp},\;\omega_{z}^{sp}\right)}^{T}$, as illustrated in Figure 6.

**Figure 7:**
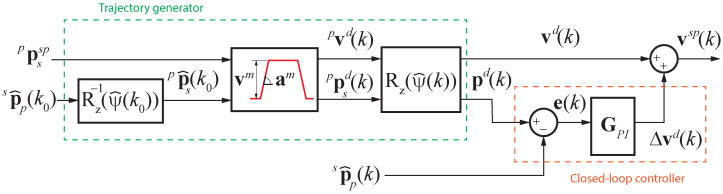
Speed set-point generation block diagram.

The speed set-points are generated using a trajectory tracking control scheme as shown in Figure 7. Given the relative pose set-point of the UAV stabilized frame w.r.t. the pattern frame at sample instant $k_{0}$, ${}^{p}p_{s}^{sp}\left(k_{0}\right)={\left({}^{p}x_{s}^{sp},\;{}^{p}y_{s}^{sp},\;{}^{p}z_{s}^{sp},\;{}^{p}\psi_{s}^{sp}\right)}^{T}$, and the estimated pose, ${}^{s}{\hat{p}}_{p}\left(k_{0}\right)$, a straight line is planned to achieve that goal, and the trajectory generator computes the desired speed and desired relative pose in the stabilized frame using a trapezoidal profile for the next sample instants *k*, $v^{d}\left(k\right)={\left(v_{x}^{d},\;v_{y}^{d},\;v_{z}^{d},\;\omega_{z}^{d}\right)}^{T}$ and $p^{d}\left(k\right)={\left(x^{d},\;y^{d},\;z^{d},\;\psi^{d}\right)}^{T}$, respectively. But firstly, ${}^{p}p_{s}^{sp}\left(k_{0}\right)$ is rotated w.r.t. z-axis by the estimated yaw angle $\hat{\psi }\left(k_{0}\right)$ to compute the trapezoidal profile in the pattern frame. Then, each desired speed and pose reference expressed in the pattern frame, ${}^{p}v_{s}^{d}\left(k\right)$ and ${}^{p}p_{s}^{d}\left(k\right)$, are rotated back to the stabilized body frame.

To compensate for disturbances and following errors, a feedback loop is added using four decoupled discrete PI controllers. So, the speed set-points sent to the flight controller are computed as

(5) $$v^{sp}\left(k\right)=v^{d}\left(k\right)+\Delta v^{d}\left(k\right),\;\;\Delta v^{d}\left(k\right)=K_{p}e\left(k\right)+K_{i}c\left(k\right),$$

where $K_{p}$ and $K_{i}$ are diagonal matrices containing the proportional and integral gains of the controllers, and the error and the cumulative error are

(6) $$e\left(k\right)=p^{d}\left(k\right)-{}^{s}{\hat{p}}_{p}\left(k\right),\;c\left(k\right)=c\left(k-1\right)+e\left(k\right),$$

respectively, with the latter initialized to zero.

**Figure 8:**
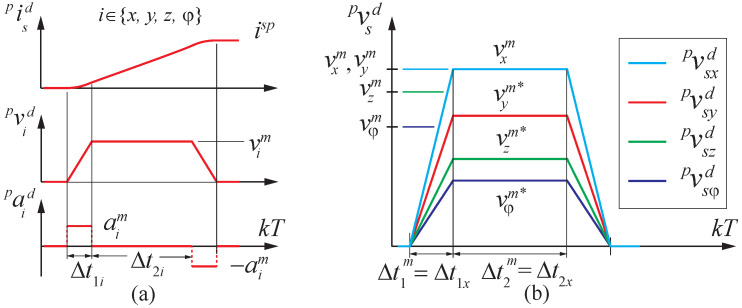
(**a**) Trapezoidal profile parameters and (**b**) speed profiles after synchronization.

The symmetric trapezoidal profile, as shown in Figure 8a, is characterized by maximum speeds and maximum accelerations $v^{m}={\left(v_{x}^{m},\;v_{y}^{m},\;v_{z}^{m},\;\omega_{z}^{m}\right)}^{T}$ and $a^{m}={\left(a_{x}^{m},\;a_{y}^{m},\;a_{z}^{m},\;\alpha_{z}^{m}\right)}^{T}$, respectively. When a set-point is commanded, the time for maximum acceleration $\Delta t_{1}={\left(\Delta t_{1x},\;\Delta t_{1y},\;\Delta t_{1z},\;\Delta t_{1\psi }\right)}^{T}$ and maximum speed $\Delta t_{2}={\left(\Delta t_{2x},\;\Delta t_{2y},\;\Delta t_{2z},\;\Delta t_{2\psi }\right)}^{T}$ segments of the profiles are computed (see Figure 8a). To ensure that the trajectory followed is a straight line, the trajectory generation should end at the same time for all axes, e.g., as shown in Figure 8b. This can be achieved by finding the maximum values $\Delta t_{1}^{m}$ and $\Delta t_{2}^{m}$, and adjusting the maximum speed and maximum acceleration of the other axes as follows:

(7) $${v_{i}}^{m★}=\frac{i^{sp}}{\Delta t_{1}^{m}},\;{a_{i}}^{m★}=\frac{i^{sp}}{\Delta t_{1}^{m}\left(\Delta t_{1}^{m}+\Delta t_{2}^{m}\right)},$$

where i∈{x,y,z,ψ}.

## 5. Results

The proposed method was tested to evaluate its effectiveness. Concretely, we performed several experiments: (i) to verify the reliability of the estimations computed with the ArUCo markers, and (ii) to evaluate the following and landing algorithms in simulation and real-world scenarios. The system configuration used regarding the localization pattern, the onboard camera and the speed set-point generator is presented next.

### 5.1. System Setup

The camera is configured to provide images with a 848×480 resolution in pixels at 30fps frame rate. The pitch and roll angles of the onboard camera frame w.r.t. the body frame are approximately equal to π/2 rad.

**Figure 9:**
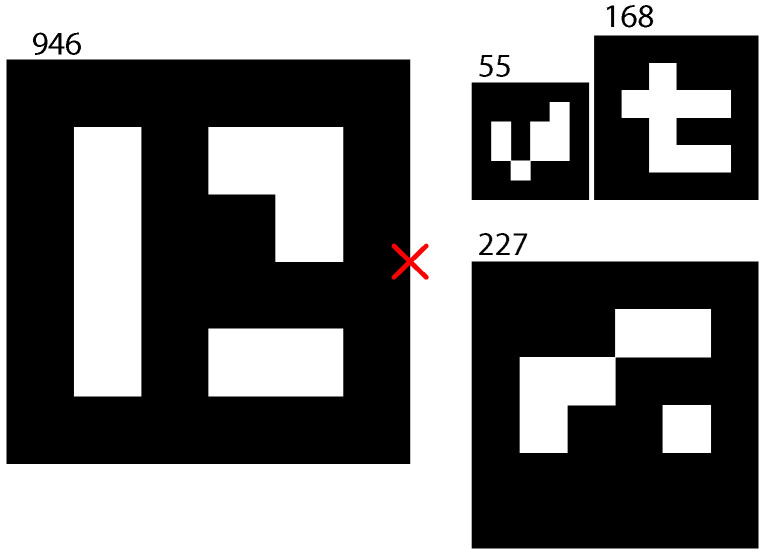
Marker pattern used for UAV/UGV relative pose estimation.

The pattern used to estimate the relative localization between the vehicles is built using markers from a 4×4 ArUco dictionary [30]. It is shown in Figure 9 with its center highlighted using a red cross. Table 1 provides the dimensions and position of the markers w.r.t to the pattern frame. The number of markers and its lengths, $L_{i}$, where selected to provide real-time robust detection at different heights. Given this pattern, the aruco_detect_node running on the Xavier NX onboard computer provides estimations of the relative pose at a maximum frequency of 14 Hz.

The parameter of the trapezoidal speed set-point trajectory generator and the PI controller gains are gathered in Table 2.

### 5.2. Pose Estimation Reliability Test

A Motion Capture System (Mo-cap) based on OptiTrack Prime 41 cameras was used to ensure that the position and heading estimations computed with the ArUCo markers were correct. The system 3D accuracy is ±0.01mm according to the manufacturer, so it can be considered to provide ground-truth measurements. For this test, the ArUcO marker pattern was fixed to the floor and several passive markers were attached to the drone to be localized with the Mo-cap system.

**Figure 10:**
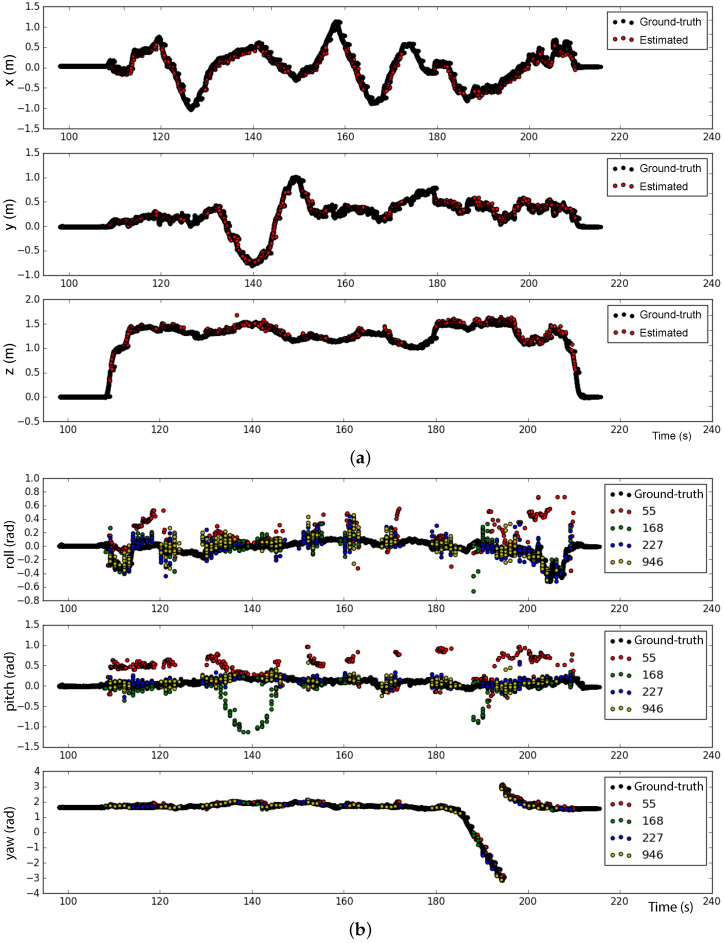
Reliability test with altitude around 1.5 m and roll and pitch angles near zero. (**a**) Position and (**b**) Euler angles estimations.

Two experiments were performed to verify the accuracy of the measurements. Firstly, the drone was moved manually in the *X* and *Y* directions at a fixed altitude without tilting. Then, at t=180 s, a full rotation w.r.t. to the drone z-axis was performed. Figure 10a shows that the average position estimated with the ArUcO markers are very close to the ground-truth provided by the Mo-Cap system. The estimated values for the yaw angle provided by each marker agree with the ground-truth as seen in Figure 10b. However, the estimated roll and pitch angles show large deviations, mainly on the smallest markers (55 and 168).

**Figure 11:**
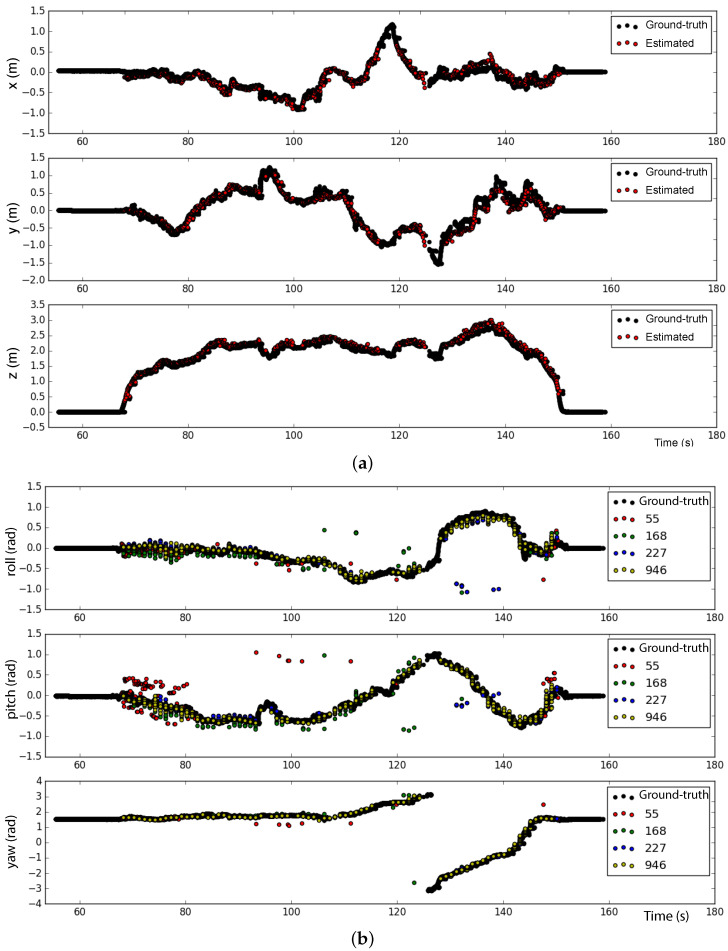
Reliability test with high values for roll and pitch angles with the drone describing a circular path. (**a**) Position and (**b**) Euler angles estimations.

In the second test, the drone was moved manually describing a circular path at around 2 m altitude centered w.r.t the marker pattern frame origin. During this motion, the drone was heavily tilted to simulate extreme flight conditions. At approximately t=110 s a rotation was applied to change its heading while tilted. As can be seen in Figure 11a,b, the ArUcO markers provide accurate estimations for the position and yaw angle, as well as, for the roll and pitch angles with the exception of some outliers.

Additional tests were conducted to find the maximum and minimum height at which the marker were detected, given as a result 4 m and 0.23 cm, respectively. Another case of markers being lost is when they fall out of the camera’s field of view, but this has not been taken into account as long as the UAV is moving faster than the UGV and the UAV is in autonomous mode, as the high-level controller will keep the markers in view when trying to follow the UGV. However, if the UAV loses the target an additional mode in the high-level controller could be implemented to move the drone faster, during a maximum amount of time, in the direction given by the last known position of the UGV Furthermore, if required, it is possible to enhance the field of view of the drone with a gimbal system or a wide-angle camera.

In view of the test results, it can be concluded that the ArUcO markers provide reliably estimation of the relative position and the yaw angle of UAV w.r.t. the marker pattern, so they can be employed to control the drone autonomously.

### 5.3. Simulation Test

**Figure 12:**
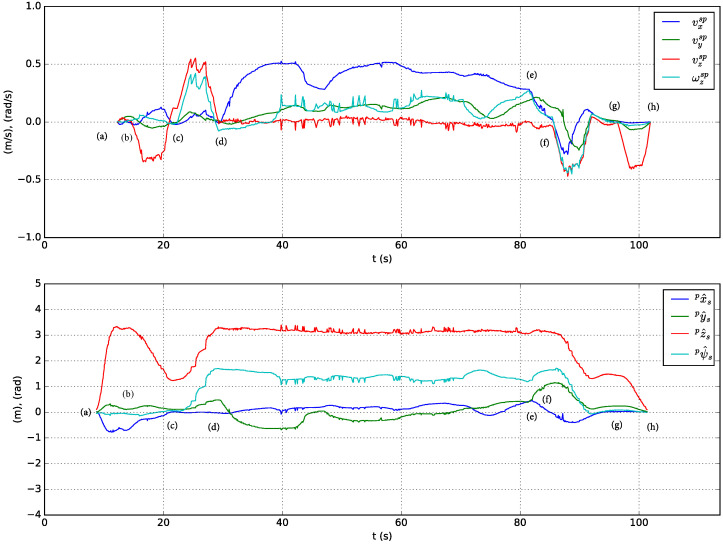
Commanded speed set-points w.r.t. the stabilized frame (**top**) and estimated relative position and heading w.r.t. the pattern frame (**bottom**) in a simulated test of the following and landing system capabilities.

**Figure 13:**
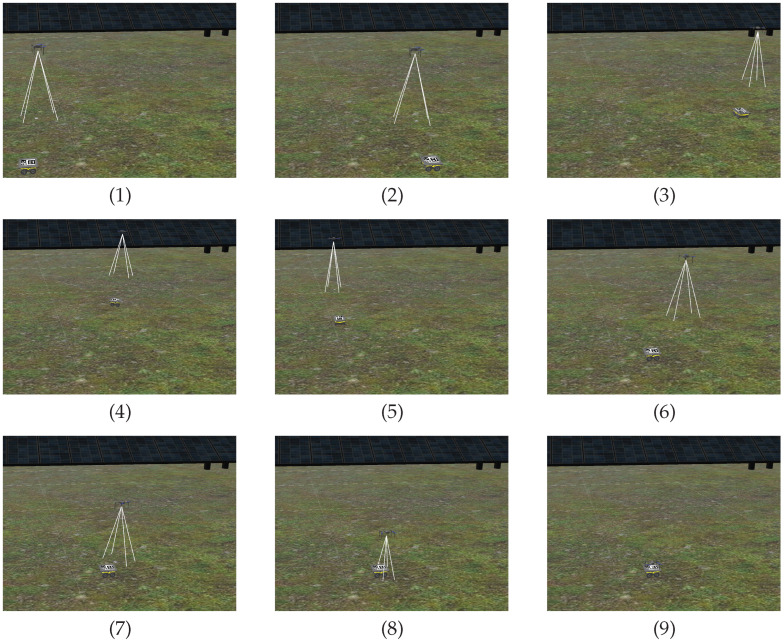
Image sequence of the following and landing test in simulation.

A sequence of following and landing actions are performed in simulation using the solar farm digital twin presented in Section 3. At the beginning of the experiment Jackal is stopped and the UGV is on top of its landing platform. Next, the actions performed are described in chronologically order and indexed with labels included in Figure 12 (top presents the speed-set point computed by high-level controller and bottom the estimated relative pose of the stabilized body frame w.r.t. the pattern frame):

(a) The UAV autopilot is in *guided* mode and it is commanded to take off and reach 3.5 m altitude.
(b) The UAV high-level controller mode is changed from teleoperation to autonomous and a set-point to approach the landing platform is commanded, ${}^{p}p_{s}^{sp}={\left(0,0,1.5\;m,0\right)}^{T}=p_{a}$. To achieve this, speed set-points with a trapezoidal profile are generated by the high-level controller (see Figure 12, top).
(c) The UAV is commanded to rotate 90${}^{\circ }$ and reach a higher location over the front side of Jackal by sending ${}^{p}p_{s}^{sp}={\left(0,0.5\;m,3\;m,\pi /2\;\mathrm{rad}\right)}^{T}=p_{f}$. Figure 13(1) shows the state of the UAV at the end of this motion.
(d) The UAV reaches the commanded set-point, see Figure 12, bottom, and Jackal starts moving describing a circular path. The PI controllers adapt the UAV speed set-points to follow Jackal maintaining the previously commanded relative pose set-point. This is illustrated in Figure 13(2)–(6).
(e) Jackal stops so the PI controllers reduced the commanded speed set-points as shown in Figure 12, top.
(f) The UAV is commanded to approach the landing platform and to rotate so it is properly aligned for landing, ${}^{p}p_{s}^{sp}=p_{a}$. Figure 13(7) shows an intermediate state of this motion.
(g) The UAV is commanded to land. In Figure 13(8) it can be seen the UAV approaching the landing platform.
(h) The UAV lands successfully as shown in Figure 13(9).

A video of this experiment can be found in the supplementary materials after conclusions.

### 5.4. Real-World Tests

**Figure 14:**
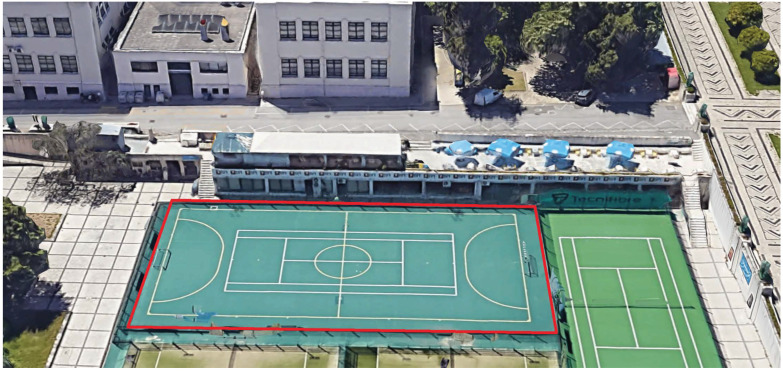
Outdoor facilities at IST Lisbon where real-world tests were conducted.

Real-world tests were conducted in the outdoor facilities of Instituto Superior Técnico (IST) in Lisbon, namely on the football court marked red in Figure 14.

**Figure 15:**
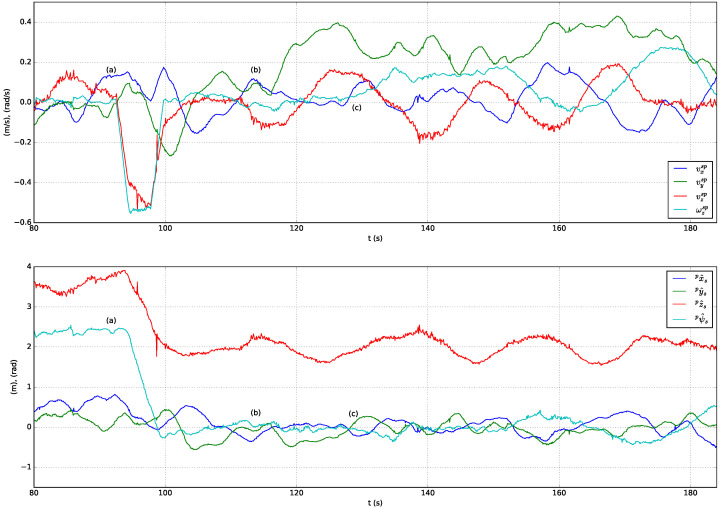
Commanded speed set-points w.r.t. the stabilized frame (**top**) and estimated relative position and heading w.r.t. the pattern frame (**bottom**) in a real-world following test.

**Figure 16:**
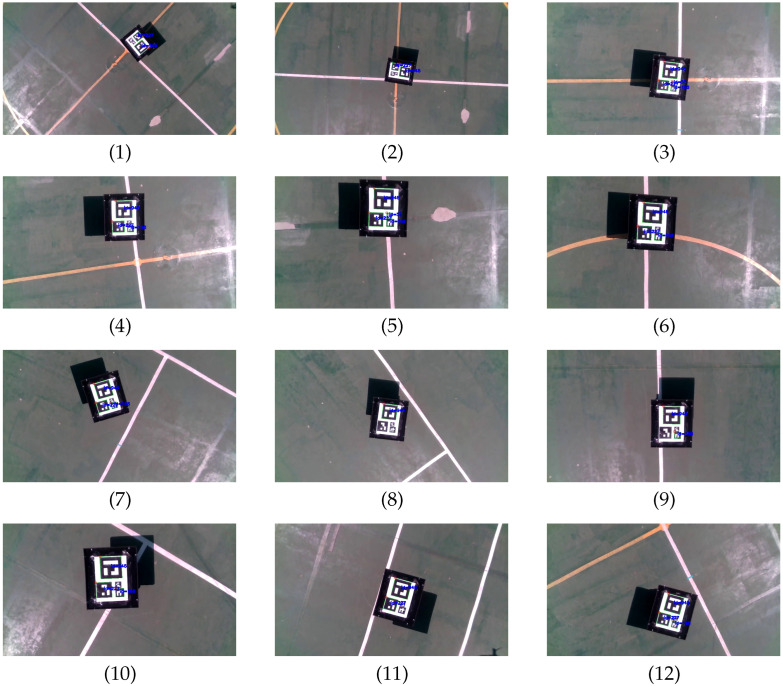
Sequence of actual following test images after processing by the UAV onboard computer.

First, following capabilities of the UAV were tested with the marker pattern attached to the landing platform of Jackal UGV. Figure 15 shows the speed set-points commanded to the UAV autopilot and the pose of the UAV w.r.t. the marker pattern estimated by the onboard computer during the test. A sequence of images captured by the onboard camera after being processed by the aruco_detect node are included in Figure 16. Prior to the test, a human operator took off the UAV and switched its mode to guided. Then, the experiment begun with the UAV in autonomous mode at an altitude of 3.5 m, and Jackal positioned in the center of the football court. The performed actions are described below using as reference the labels included in Figure 15.

(a) A relative pose set-point is commanded to center the UAV on top of Jackal at 2 m altitude, ${}^{p}p_{s}^{sp}={\left(0,\;0,\;2\;m,\;0\right)}^{T}$, so speed set-points to reduce the altitude and the heading are generated as shown in Figure 15, top. At approximately at t=100 s the relative pose set-point is reached and it is maintained by the PI controllers (Figure 15, bottom). The images captured by the onboard camera while the UAV is descending and rotating are presented in Figure 16(1)–(3).
(b) Jackal is teleoperated to move forward (see Figure 16(4)–(6)), so the PI controllers begin to increase $v_{y}^{sp}$ to maintain the commanded relative pose and follow Jackal, as shown in Figure 15b.
(c) Jackal is commanded to rotate to its left, as shown in Figure 16(7),(8)), so $\omega_{z}^{sp}$ is increased by the UAV high-level controller (Figure 15(c), top). Next, a sequence of forward and left turn commands lead Jackal to the initial test position while the UAV autonomously follows it (see Figure 16(9)–(12)).

**Figure 17:**
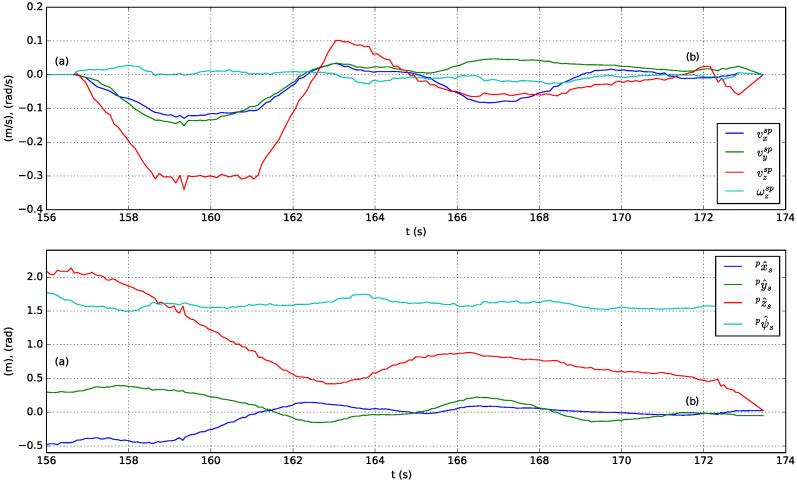
Commanded speed set-points w.r.t. the stabilized frame (**top**) and estimated relative position and heading w.r.t. the pattern frame (**bottom**) in a real-world landing test.

**Figure 18:**
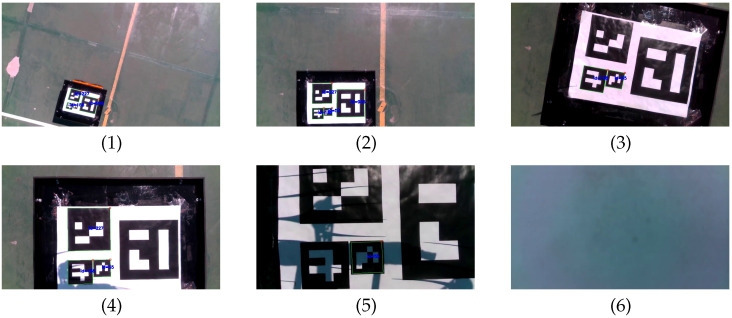
Sequence of actual landing test images after processing on board the UAV.

The autonomous landing experiment begins with Jackal stopped at the center of the football court, while the UAV is in autonomous mode at 2 m height over the UAV, but not centered. The performed actions are described below using as reference the labels included in Figure 17:

(a) The UAV is commanded to approach Jackal for landing by sending the set-point, ${}^{p}p_{s}^{sp}={\left(0,\;0,\;5\;m,\;\pi \;\mathrm{rad}\right)}^{T}$. The computed speed set-points reduces the altitude and center the UAV over the landing platform as shown in Figure 17, bottom, and Figure 18(1)–(4).
(b) The position and heading errors are detected to be small enough (see Figure 17, bottom, and Figure 18(5)) so the UAV is commanded to land. The set-point $v_{z}^{sp}$ is reduced and the UAV lands on Jackal’s platform (see Figure 18(6)).

Although there were external disturbances such as wind, which reduced the accuracy of the high-level controller compared to its simulated counterpart, the UAV was able to follow the Jackal UGV and land on its platform successfully.

Videos of these experiments can be found in the supplementary materials after conclusions.

## 6. Conclusions

This work has presented a vision-based method that allows an aerial robot to autonomously follow and land on a ground mobile platform. This approach uses a custom-designed landmark pattern based on ArUCo markers as a guiding system for the UAV, which has been validated using a Mo-cap system. Unlike other implementations, it relies exclusively on the markers for following and landing. The developed system accepts relative position and heading set-points between the UAV and the UGV, which are reached by planning straight line segments from the current UAV location. The UAV controller has been implemented using a hierarchical structure: an Ardupilot-based commercial flight controller has been used as the low-level controller, while the high-level controller has been implemented in the UAV onboard computer using ROS. The low-level controller accepts UAV speed set-points computed by the high-level controller using a trajectory control scheme, with a trapezoidal profile speed generator as feedforward and four decoupled PI controllers in the feedback loop.

The proposed framework has been tested in simulation and real environments using the digital twin of a solar farm and at the outdoor facilities provided by ISR Lisboa, respectively. In both scenarios, the UAV has been able to autonomously follow, with a specific relative pose, a teleoperated ground mobile robot equipped with a landing platform and a marker pattern on top, as well as to land on it when commanded to do so.

A possible future line of work is to investigate the scalability of the proposed approach for scenarios with several aerial and ground mobile robots operating simultaneously. This could involve the development of new algorithms and protocols to enable coordination and collaboration between robots to tackle more complex tasks.

## Figures and Tables

**Table 1:** Localization pattern parameters.

Id	$L_{i}$ (m)	${}^{p}x_{i}$ (m)	${}^{p}y_{i}$ (m)
55	0.06	−0.06175	0.06175
168	0.084	−0.0738	0.1367
227	0.147	0.0735	0.1053
946	0.207	0	−0.1035

**Table 2:** Parameters of the trajectory generator and the PI controllers gains.

	$v^{m}$	$a^{m}$	$k_{p}$	$k_{i}$
	**(m s** ${}^{-1}$ **)**	**(m s** ${}^{-2}$ **)**	**(s** ${}^{-1}$ **)**	**(s** ${}^{-1}$ **)**
*x*	0.8	0.4	0.4	0.01
*y*	0.8	0.4	0.4	0.01
*z*	0.35	0.17	0.4	0.01
	($\mathrm{rad}\;s^{-1}$)	($\mathrm{rad}\;s^{-2}$)	($s^{-1}$)	($s^{-1}$)
ψ	0.5	0.25	0.5	0.02

## Supplementary Materials

The videos of the simulated following and landing test as well as the corresponding real-world tests are linked https://www.uma.es/robotics-and-mechatronics/info/138139/diomedes/?set_language=en#videos-tests.

## Abbreviations

The following abbreviations are used in this manuscript:

GNSS	Global Navigation Satellite System
GPS	Global Positioning System
IMU	Inertial Measurement Unit
UAV	Unmanned Aerial Vehicle
UGV	Unmanned Ground Vehicle
SiL	Software-in-the-Loop
SBC	Single Board Computer
ROS	Robot Operating System
w.r.t.	with respect to
